# Anti-apoptotic response during anoxia and recovery in a freeze-tolerant wood frog (*Rana sylvatica)*

**DOI:** 10.7717/peerj.1834

**Published:** 2016-03-24

**Authors:** Victoria E.M. Gerber, Sanoji Wijenayake, Kenneth B. Storey

**Affiliations:** 1 Department of Biology, Carleton University, Ottawa, ON, Canada; 2 Institute of Biochemistry, Department of Biology and Chemistry, Carleton University, Ottawa, ON, Canada; 3 Current affiliation: Faculty of Medicine, University of Ottawa, Ottawa, ON, Canada

**Keywords:** Anoxia, Wood frog, Pro-survival, Apoptosis, Cytoprotection, Hypometabolism

## Abstract

The common wood frog, *Rana sylvatica*, utilizes freeze tolerance as a means of winter survival. Concealed beneath a layer of leaf litter and blanketed by snow, these frogs withstand subzero temperatures by allowing approximately 65–70% of total body water to freeze. Freezing is generally considered to be an ischemic event in which the blood oxygen supply is impeded and may lead to low levels of ATP production and exposure to oxidative stress. Therefore, it is as important to selectively upregulate cytoprotective mechanisms such as the heat shock protein (HSP) response and expression of antioxidants as it is to shut down majority of ATP consuming processes in the cell. The objective of this study was to investigate another probable cytoprotective mechanism, anti-apoptosis during oxygen deprivation and recovery in the anoxia tolerant wood frog. In particular, relative protein expression levels of two important apoptotic regulator proteins, Bax and p-p53 (S46), and five anti-apoptotic/pro-survival proteins, Bcl-2, p-Bcl-2 (S70), Bcl-xL, x-IAP, and c-IAP in response to normoxic, 24 Hr anoxic exposure, and 4 Hr recovery stages were assessed in the liver and skeletal muscle using western immunoblotting. The results suggest a tissue-specific regulation of the anti-apoptotic pathway in the wood frog, where both liver and skeletal muscle shows an overall decrease in apoptosis and an increase in cell survival. This type of cytoprotective mechanism could be aimed at preserving the existing cellular components during long-term anoxia and oxygen recovery phases in the wood frog.

## Introduction

Winter survival usually depends on an animal’s ability to utilize physiological and biochemical mechanisms of freeze tolerance, freeze avoidance, or migration. Complete freezing of bodily fluids can be lethal to most animals and thus animals that use freeze-avoidance strategies substantially decrease the supercooling point of their bodily fluids and employ anti-freezing strategies to prevent spontaneous nucleation at subzero temperatures (Storey & Storey, 1988). By contrast, freeze-tolerant animals allow freezing of the extracellular fluid and utilize extracellular ice nucleating agents (INA) to manage ice growth but stringently prevent freezing of the cytoplasmic fluid (Storey & Storey, 2004a). Natural freeze tolerance is known to occur in terrestrial insects of the Coleoptera, Diptera, Hymenoptera, and Lepidoptera orders (Ring, 1980; Block, 1982; Storey & Storey, 1988), intertidal marine gastropods and barnacles (Aarset, 1982; Murphy, 1983), and several species of terrestrial frogs, one of which is the freeze-tolerant wood frog, *Rana sylvatica* (Storey & Storey, 1985; Storey & Storey, 1988).

The North American wood frog, *R. sylvatica*, has the remarkable ability to survive winter temperatures that fall below 0 °C by converting 65–70% of total body water into extracellular ice (Storey & Storey, 1984; Storey & Storey, 2004a), and has been categorized as an “ecologically relevant” freeze tolerant species because unlike most other vertebrates, it has the ability to endure long-term freezing (days to weeks at a time) with a stable maximum ice content at subzero temperatures. The wood frog endures the penetration of ice crystals to a majority of their extracellular spaces including the brain, lungs, heart, abdominal cavity, plasma, bladder, and eye lens that interfere with all vital physiological functions such as neuronal activity, muscle movement, breathing, circulation, digestion and metabolism, and waste filtration (Layne & First, 1991; Kling, Costanzo & Lee, 1994; Storey & Storey, 2004b). Ice formation in the extracellular spaces can create many physiological concerns for the organism; one of which is the extensive damage crystal growth can cause to subcellular tissues and organs. Ice formation can burst capillary walls (Rubinsky et al., 1987), induce movement of inner organs, as well as produce reactive oxygen species (ROS) during recovery (Storey & Storey, 2004a; Storey & Storey, 2004b).

In addition, prolonged exposure to subzero temperatures can directly affect metabolic processes in several ways; (1) Conformation, orientation, and fluidity of the membrane phospholipids are temperature sensitive and thus can affect binding of transmembrane proteins, diffusion and transport, and proper functioning of membrane-bound metabolic processes. (2) Exposure to low temperature outside of the optimal range can induce protein denaturation that not only affect the functioning of individual metabolic enzymes but can also affect proper functioning of integrative metabolic pathways, the formation of multi-enzyme complexes, protein-protein interactions, and post-translational modifications of regulatory proteins (Storey & Storey, 1988).

Another main consequence of freezing is the onset of anoxia (lack of oxygen) and ischemia (disruption of oxygen flow via blood) (Hochachka, 1986). Freezing in a nutshell should be thought of as an ischemic event (Storey & Storey, 2004b). Extracellular freezing thickens and solidifies the blood plasma and hinders the delivery of oxygen, fuels, nutrients, and hormones to cells and organs. Prolonged exposure to subzero temperatures and low levels of oxygen circulation can generate an imbalance between ATP utilization and ATP production. If the organism under anoxia is no longer using mitochondrial oxidation to generate ATP to its fullest capacity, yet continue to utilize the available ATP at a normoxic rate, fuel reserves will diminish in a very short amount of time. ATP-limited frozen state cannot withstand regular rates of metabolic activity and as such *R. sylvatica* has been shown to downregulate its metabolic rate by suppressing energy consuming cellular processes such as gene expression (Storey, 2004), protein synthesis and degradation, cell cycle, ATP motive ion channel function, and selectively regulate the activities and expression levels of 25–28 metabolic enzymes that participate in glycolysis, TCA cycle, gluconeogenesis, amino acid catabolism, and fatty acid oxidation (Storey & Storey, 1990; Cowan & Storey, 2001; Storey & Storey, 2004b). Furthermore, ischemic-reperfusion events that are associated with the freeze-thaw cycles in the wood frog produce reactive oxygen species (ROS) and can be a major source of oxidative stress (Storey & Storey, 2011). As such, several antioxidant enzymes have been shown to increase in activity during anoxia and reperfusion in the common wood frog (Joanisse & Storey, 1996). Other anoxia-tolerant vertebrates such as the red-eared sliders (*Trachemys scripta elegans*) utilize a myriad of cell preservation strategies such as the heat shock protein response (HSPs), unfolded protein response (UPR), and upregulation of antioxidant defenses to combat oxidative stress even though the overall metabolic rate of the animal has been reduced to approximately 10% of the normoxic values (Krivoruchko & Storey, 2010; Krivoruchko & Storey, 2015). This could suggest that anoxia-responsive signal transduction pathways may regulate the interplay between the hypometabolic and cytoprotective responses during anoxia and reperfusion in a stringent manner (Cowan & Storey, 2003), and not all cellular processes are universally shut down but rather selectively up and downregulated to meet the cellular demands of the organism (Cowan & Storey, 2001).

Although much work has been done on the physiological and biochemical responses to long-term freezing in wood frogs, the balance between cell survival and cell death (apoptosis) and the use of anti-apoptosis as a possible cytoprotective mechanism has not been studied. Previous work done on the champion mammalian hibernator, the 13-line ground squirrel (*Ictidomys tridecemlineatus)* have illustrated that anti-apoptosis may be used as a cytoprotective mechanism to preserve cellular integrity during torpor-arousal cycles (Rouble et al., 2013). Furthermore, hibernation studies done in the brain of horseshoe bats have discovered multiple genes that are associated with apoptosis to be significantly upregulated (Chen et al., 2008).

Exploring the cytoprotective role of the anti-apoptotic pathway in a naturally occurring freeze-tolerant model such as the wood frog has tremendous therapeutic potential, as important regulatory mechanisms that enable stress-tolerant species to survive are usually dysfunctional in humans illnesses and/or potential hurdles to overcome in cryopreservation of organs. Previous studies have shown substantial overlap between the stressors that are associated with cryopreservation and stressors that are known to activate apoptosis (such as freeze/thaw and anoxia/recovery) and failures associated with cryopreservation have now been directly linked to apoptosis (Baust, Van Buskirk & Baust, 1998; Baust, Dayong & Baust, 2009).

Kerr, Wyllie & Currie (1972) characterized apoptosis initially as plasma membrane blebbing, cell shrinkage, chromatin condensation, and degradation of DNA. Apoptosis is now recognized to be a tightly controlled and actively regulated process of cell death, and plays a vital role in cellular immunity as well as in the regulation of cellular growth and differentiation (Elmore, 2007). There are two main apoptotic pathways—extrinsic and intrinsic, and both pathways receive information and activate apoptosis independently of one another (Ashkenazi, 2008).

**Figure 1:**
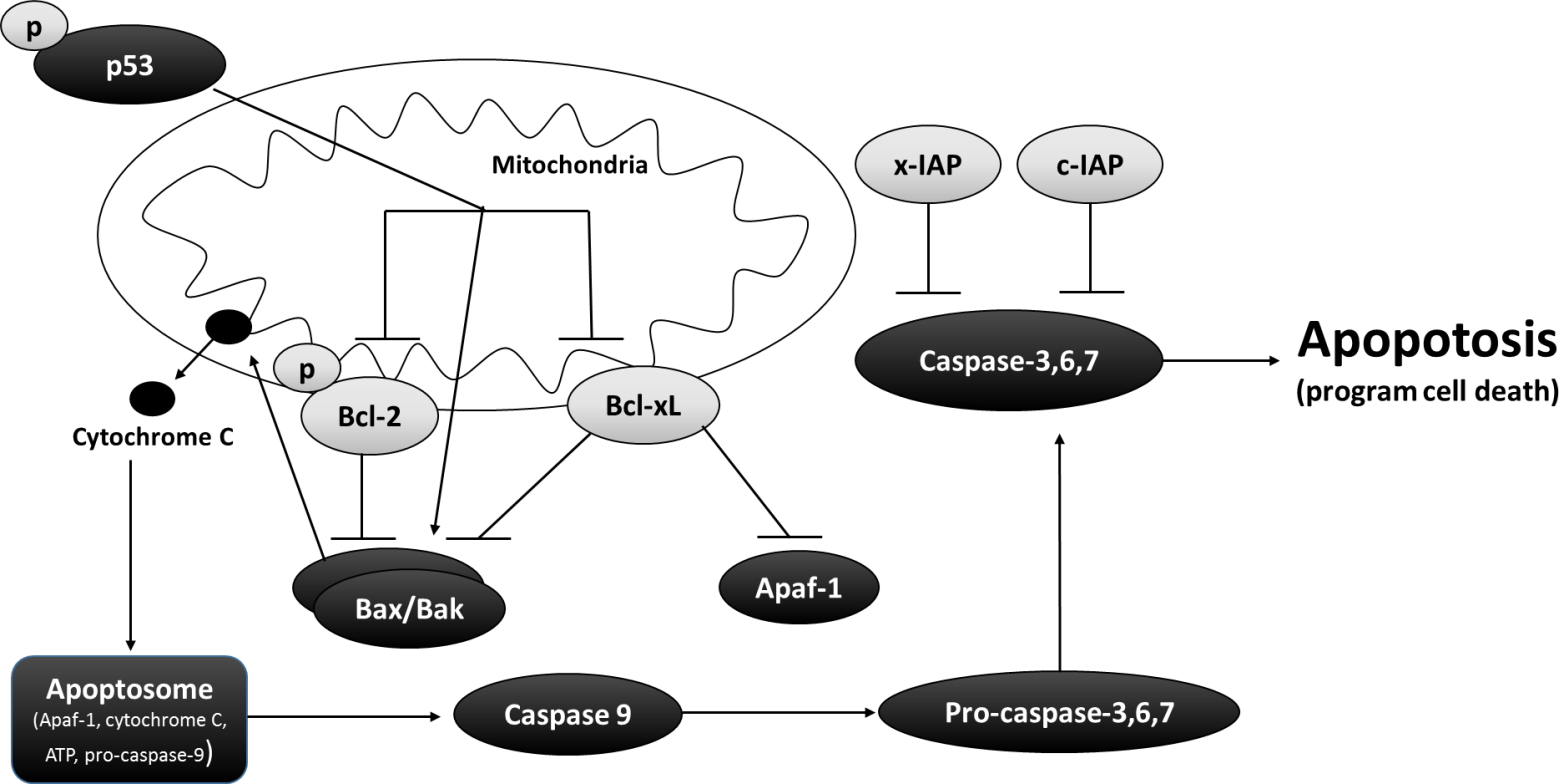
Interrelationship between pro and anti-apoptotic proteins in the mitochondrial matrix. Bcl-2 and Bcl-xL are two well-known anti-apoptotic proteins that protects the integrity of the mitochondrial matrix by suppressing Bax, Apaf-1, cytochrome c, and Caspase 9 from initiating programmed cell death during times of low cellular energy. In addition, x-IAP and c-IAP although not directly associated with the functioning of the mitochondrial matrix, inhibit pro-apoptotic proteins such as Caspase 3, 6, and 7. The arrow head designate pro-regulation while blunt-head designates inhibitory regulation. The image was modified from Rouble et al. (2013).

The extrinsic apoptotic pathway is activated from stimuli outside the cell by apoptosis inducing ligands (e.g., growth factors, hormones, cytokines, toxins, etc.). However, the primary focus of this paper is the intrinsic (or mitochondrial) apoptotic pathway, which activates apoptosis from inside the cell through interactions between members of the B-cell leukemia/lymphoma (Bcl) protein family. The Bcl family is a superfamily that can be divided to three subfamilies; one promoting cell death (ex. Bax and Bak), another promoting cell survival or anti-apoptosis (ex. Bcl-2 and Bcl-xL), and a third family with a conserved BH3 domain (ex. Bid and Bim) enabling them to bind and regulate anti-apoptotic Bcl-2 proteins. Developmental cues or severe cellular stress will stimulate the intrinsic pathway through transcription or post-translational activation of activator BH3-only proteins (Bid and Bim), which in turn drive the activation of pro-apoptotic proteins (Bax and Bak) (Xu et al., 2013). Studies into the biochemical, cellular, and physiological roles of Bcl family members have revealed that BH3-only proteins displace anti-apoptotic proteins Bcl-2 and Bcl-xL to allow pro-apoptotic proteins (Bax and Bak) to self-associate and engage the mitochondria (Fig. 1) (Kodama et al., 2012; Xu et al., 2013).

p53 is a well characterized tumor suppressor and cell cycle regulatory protein that is also stabilized and activated by cellular stress (Oren, 1994; Ko & Prives, 1996; Levine, 1997). p53 is responsible for initiating several cellular responses such as cell cycle arrest, senescence, cellular differentiation, and repair of genotoxic damage. Mechanisms of p53-dependent apoptosis include p53 inducing Bax and Bak oligomerization and physically interacting with Bcl-xL and Bcl-2 to antagonize their anti-apoptotic effects (Wolff et al., 2008). Notably, phosphorylation of p53 at S46 residue has been shown to induce apoptosis (Oda et al., 2000; Vousden & Lu, 2002) by regulating the mitochondrial release of cytochrome c and SMAC (Schuler & Green, 2001), and increasing the expression of genes that inhibit cell survival (Bouvard et al., 2000).

Inhibitor of apoptotic proteins (IAPs), c-IAP and x-IAP, impede apoptosis at the level of effector caspase activation. IAPs will suppress intracellular proteases that facilitate the proteolytic cleave and activation of effector caspases (Deveraux & Reed, 1999). In particular, IAPs block cell death by binding and inhibiting effector caspases-3 and caspace-7, as well as suppress intercommunication between intrinsic and extrinsic apoptotic pathways.

The current study looks at whether programmed cell death (apoptosis), considered to be an energy expensive process, is selectively regulated during anoxia exposure in the freeze-tolerant wood frog and whether an anti-apoptosis response may play a cytoprotective role to minimize cellular damage. In particular, the expression patterns of two important apoptotic regulator proteins, Bax and p-p53 (S46), and five anti-apoptotic/pro-survival proteins, Bcl-2, p-Bcl-2 (S70), Bcl-xL, c-IAP, and x-IAP, in response to 24 Hr anoxic exposure and 4 Hr recovery stages were analyzed in the liver and skeletal muscle of *R. sylvatica*.

## Material and Methods

### Animal preparation

During early spring, active and mature male wood frogs were sampled from meltwater ephemeral ponds in lightly wooded areas near Oxford Mills, Ontario, Canada on the first or second night when breeding choruses were active in mid-April. Water temperature was about 5–7 °C. Frogs were held in coolers on snow taken from the edges of the ponds and transported to the laboratory. Prior to experimentation, the frogs were washed in a tetracycline bath and then—kept at 5 °C for 1–2 weeks inside plastic containers with damp sphagnum moss flooring before proceeding to anoxic treatments. Control (normoxic) frogs were sampled directly from this condition. Note: 5 °C is the average seasonal temperature recorded in Ottawa during early spring and the frogs remained active after being kept at 5 °C for 1–2 weeks.

For the anoxia experiments, 5 °C acclimated adult frogs were placed in sealed plastic jars (700 mL) with a layer of damp paper towels (wetted with distilled water that was previously bubbled with 100% N_2_ gas) and were chilled on crushed ice. The jars used were connected to a nitrogen gas line by a syringe port on the lids. The jars were flushed with nitrogen gas for 20 min (a second port allowed air to vent). Frogs were quickly placed in the jars (6–8 frogs per jar), all ports were closed using parafilm, and nitrogen gassing was continued for 30 min at 5 °C. Jars were then placed in a 5 °C incubator for 24 Hrs. After 24 Hr of anoxic exposure, half the jars were placed on ice and the nitrogen lines were reconnected. The 24 Hr anoxic frogs were sampled from this condition. Frogs in the remaining jars were transferred to new jars and exposed to normal air for 4 Hr at 5 °C to facilitate aerobic recovery and sampled immediately. A 24 Hr anoxia exposure was chosen based on a previous demonstration by Holden & Storey (1997) that showed wood frogs can readily endure as much as 48 Hr anoxia at 5 °C (Note: 30 mins, 1 Hr, 4 Hr, and 48 Hr anoxic exposure was conducted without mortality in any experimental group). Furthermore, the use of the 24 Hr anoxic exposure time and 4 Hr recovery time points parallels several published studies of the metabolic responses to anoxia by wood frogs (Zhang & Storey, 2012; Zhang & Storey, 2013; Sullivan, Biggar & Storey, 2015a) as well as studies of freeze/thaw response in the wood frog (McNally et al., 2002; McNally, Sturgeon & Storey, 2003; Sullivan, Biggar & Storey, 2015b). All frogs were sacrificed by pithing, and liver and skeletal muscle of the hind legs from control, 24 Hr anoxia, and 4 Hr recovery were dissected and immediately placed in liquid nitrogen andsubsequently stored at −80 °C for future use.

All animal protocols for care, experimentation, and euthanasia had the approval of the Carleton University Animal Care Committee (#13683) in accordance with the guidelines established by the Canadian Council on Animal Care. Note that 24 hr anoxic stress is survivable in nature by the wood frogs.

### Total protein isolation

Briefly, frozen samples were quickly weighed, crushed into small pieces under liquid nitrogen using a mortar and pastel. Frozen tissues were homogenized 1:2 w/v in pre-chilled homogenizing buffer (20 mM HEPES, pH 7.4, 200 mM NaCl, 0.1 mM EDTA, 10 mM NaF, 1 mM Na_3_VO_4_, 10 mM *β*-glycerophosphate), 1 mM phenylmethylsulfonyl fluoride (PMSF) and 1 µl/ml Protease Inhibitor Cocktail (BioShop; Cat. # PIC001) using a Polytron PT10 homogenizer. Supernatants were removed after the samples were centrifuged at 12,000 rpm for 15 min at 4 °C. The Bradford assay was used to measure the soluble protein concentrations using the Bio-Rad protein reagent (BioRad Laboratories, Hercules, CA; Cat # 500–0006) and a MR5000 microplate reader at 595 nm. Final protein concentrations were adjusted to 10 µg/µL for controls and liver stress samples and 5 µg/µL for skeletal muscle stress samples by adding a small volume of homogenizing buffer. Samples were then mixed 1:1 v/v with 2X SDS sample buffer (100 mM Tris-base, 4% w/v SDS, 20% v/v glycerol, 0.2% w/v bromophenol blue, 10% v/v 2-mercaptoethanol, pH 6.8), boiled for 5 min and immediately stored at −40 °C until use.

### Immunoblotting

SDS-polyacrylamide gels were prepared containing 0.13 M Tris pH 6.8, 0.1% SDS, 0.1% APS, 0.1% TEMED and acrylamide concentrations of 5% for the upper stacking gel and 8–15% for the resolving gel depending on the molecular weight of the protein of interest. Aliquots of wood frog liver or skeletal muscle samples containing 20–30 ug of soluble protein were loaded into wells and resolved on a BioRad Mini Protean III apparatus at 180 V for 60–90 min. In order to confirm the molecular weight of the target protein bands, 3–5 ul aliquots of PiNK Plus Prestained Protein Ladder (Froggabio: PM005-0500) were loaded onto one of the lanes.

Proteins were then electroblotted onto 0.25 um polyvinylidene difluoride (PVDF) membranes by wet transfer with 1X transfer buffer (25 mM Tris pH 8.5, 192 mM glycine, 20% methanol) at 4 °C for 90 min at 160 mA and blocked with high molecular weight polyvinyl alcohol (PVA) (70–100 kDa) or milk (2.5–10% w/v) made up in 0.5% TBST solution (20 mM Tris base, pH 7.6, 140 mM NaCl, 0.05% v:v Tween-20). Membranes were then probed with specific primary antibodies (1:1,000 dilution in 0.5% TBST) at 4 °C for 12–24 Hrs on a rocking platform. Antibodies each cross-reacted with single strong bands on the immunoblots at the expected molecular masses for Bcl-2 (MW: 26 kDa—Cat #2870), p-Bcl-2 (MW: 26 kDa—Cat #2827), Bcl-xL (MW: 30 kDa—Cat #2764), c-IAP (MW: 69 kDa—Cat #4952), and p-p53 (S46) (Mw: 40 kDa—Cat#2521) were purchased from Cell Signaling). x-IAP (53 kDa) (Cat #7074) was purchased from Santa Cruz and Bax (24 kDa) (Cat #GTX77804) was purchased from GeneTex.

Membranes were washed several times with 0.5% TBST, followed by incubation with horse radish peptidase (HRP)-linked anti-rabbit IgG secondary antibody (1:8000 v/v dilution) for ∼30 min before visualization using enhanced chemiluminescence (H_2_O_2_ and luminol). Blots were then stained using Coomassie blue stain (0.25% w/v Coomassie brilliant blue, 7.5% v:v acetic acid, 50% methanol).

### Data and statistics

ChemiGenius Bio Imaging System and GeneTools software (SynGene) were used to visualize and quantify the immunoblots respectively. Bands were normalized against several Coomassie blue stained protein bands with constant expression between control and 24 Hr anoxic and 4 Hr recovery conditions and are of different molecular weight from the target band. Data were expressed as means ± SEM, *n* = 3–4 independent samples from different animals and were analyzed using one-way analysis of variance followed by a Tukey Post-Hoc test; *p* < 0.05 was accepted as indicating a statistically significant difference.

## Results

### Anti/Pro-apoptosis response in liver tissue of anoxia tolerant wood frog

**Figure 2:**
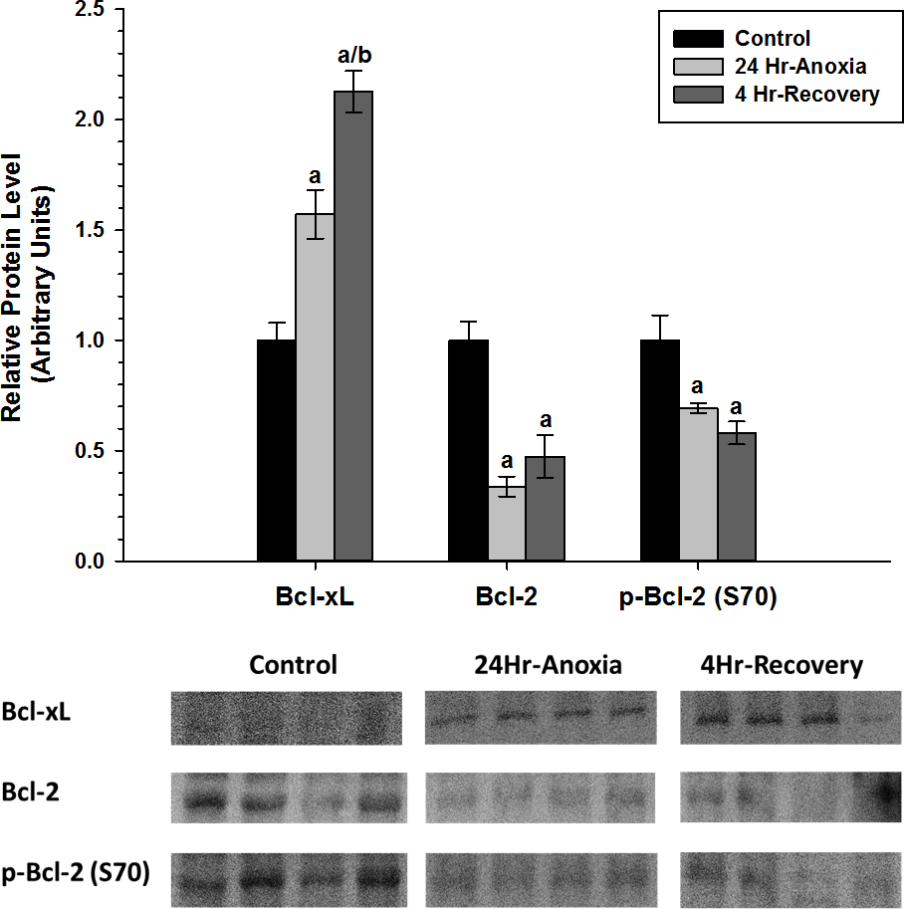
Relative changes in the protein expression levels of the anti-apoptotic Bcl family of proteins (Bcl-2, p-Bcl-2 (S70), and Bcl-xL) in the liver of *Rana sylvatica* in response to 24 Hr anoxia and 4 Hr recovery as determined by western immunoblotting. Data are mean ± SEM (*N* = 3–5 independent protein isolations from different animals). Data were analyzed using analysis of variance with a post hoc Tukey test (*p* < 0.05); (A) Significantly different from the corresponding control (*P* < 0.05). (B) Significantly different values from 24 Hr anoxia.

Relative protein expression levels of anti-apoptotic proteins Bcl-2, p-Bcl-2 (S70), and Bcl-xL were measured using western immunoblotting in liver tissues (Fig. 2). Bcl-xL exhibited 1.5-fold upregulation in response to 24 Hr anoxia and 2.0-fold upregulation during 4 Hr recovery compared to normoxic control conditions. Bcl-2 activity is known to be regulated by site-specific phosphorylation at S70 residue, and both phosphorylated and non-phosphorylated forms of Bcl-2 showed decreased expression compared to the normoxic condition (*p* < 0.05).

**Figure 3:**
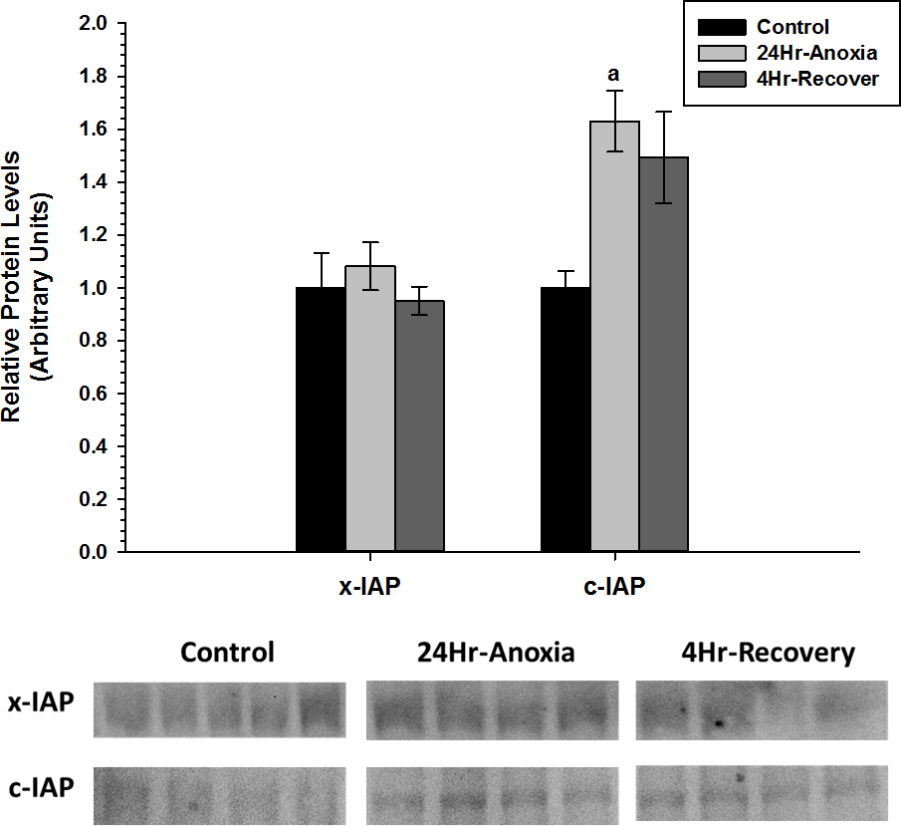
Relative changes in the protein expression levels of the anti-apoptotic proteins x-IAP and c-IAP in the liver of *Rana sylvatica* in response to 24 Hr anoxia and 4 Hr recovery as determined by western immunoblotting. Data are mean ± SEM (*N* = 3–5 independent protein isolations from different animals). Other information as in Fig. 2.

The relative expression levels of the two anti-apoptotic proteins, x-IAP and c-IAP that are responsible for inhibiting the function of Caspase 3, 6, 7 were also assessed using western immunoblotting (Fig. 3). x-IAP showed no change in expression, whereas c-IAP showed 0.6-fold increase in expression in response to 24 Hr anoxia (*p* < 0.05).

**Figure 4:**
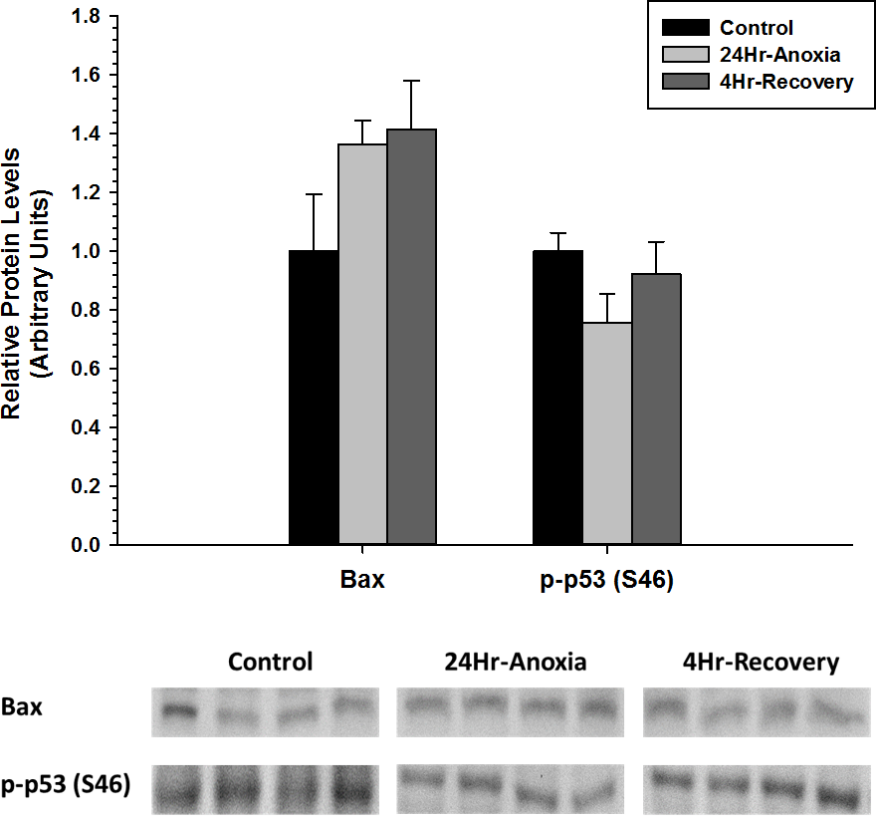
Relative changes in the protein expression levels of the pro-apoptotic proteins Bax and p-p53 (S46) in the liver of *Rana sylvatica* in response to 24 Hr anoxia and 4 Hr recovery as determined by western immunoblotting. Data are mean ± SEM (*N* = 3–5 independent protein isolations from different animals). Other information as in Fig. 2.

Furthermore, relative protein expression levels of pro-apoptotic proteins Bax and p-p53 (S46) were measured using western immunoblotting in liver tissues (Fig. 4). When posttranslational modified at S46 residue, p53 facilitates pro-apoptosis and Bax function in its unmodified form as a pro-apoptotic factor. Both Bax and p-p53 (S46) showed no differential expression upon exposure to 24 Hr anoxia and 4 Hr recovery conditions.

### Anti/Pro-apoptosis response in skeletal tissue of anoxia tolerant wood frog

**Figure 5:**
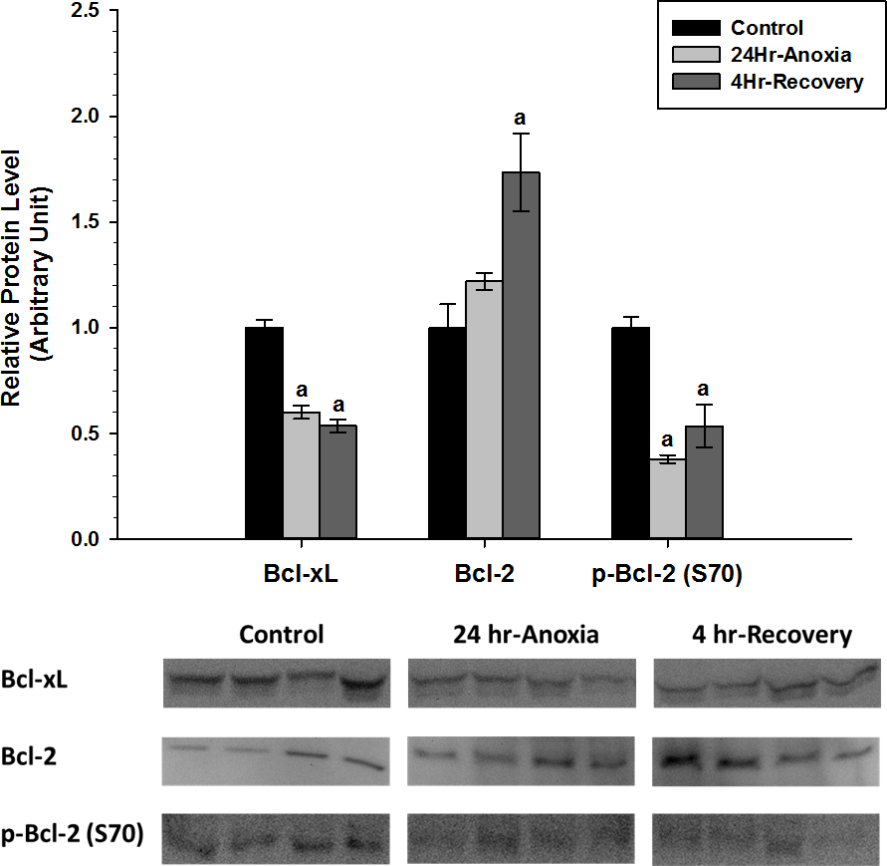
Relative changes in the protein expression levels of anti-apoptotic Bcl family of proteins (Bcl-2, p-Bcl-2 (S70), and Bcl-xL) in the skeletal muscle of *Rana sylvatica* in response to 24 Hr anoxia and 4 Hr recovery as determined by western immunoblotting. Data are mean ± SEM (*N* = 3–5 independent protein isolations from different animals). Other information as in Fig. 2.

**Figure 6:**
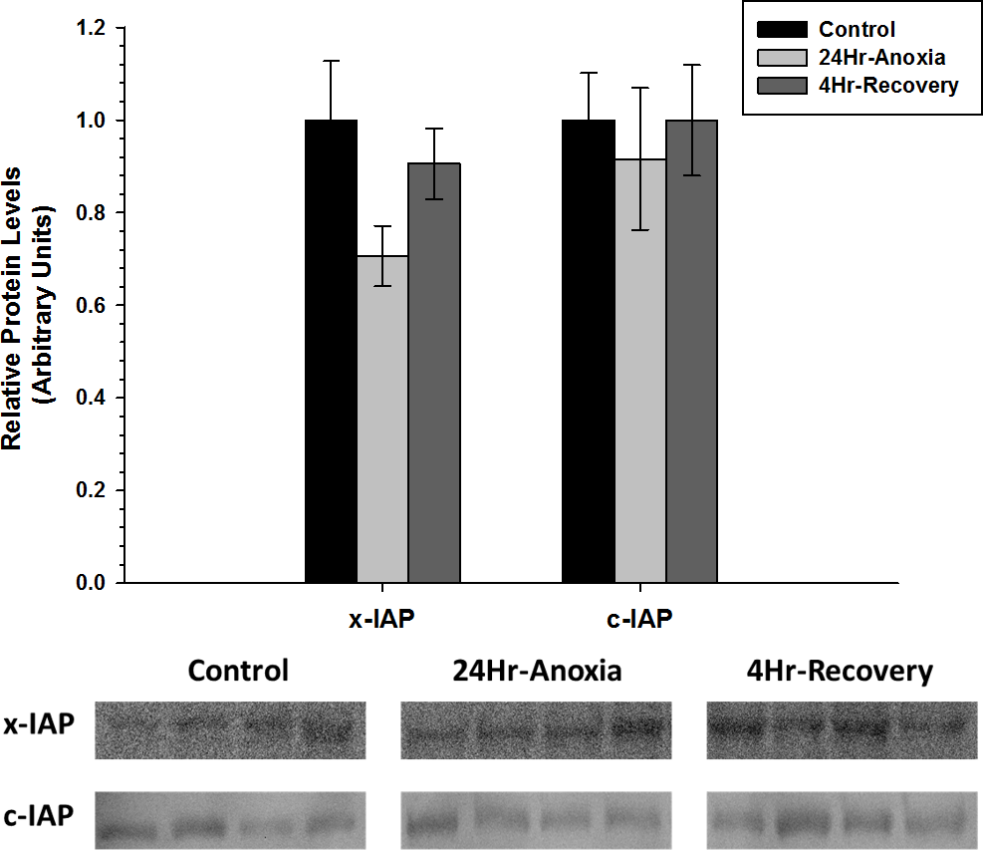
Relative changes in the protein expression levels of the anti-apoptotic proteins x-IAP and c-IAP in the skeletal muscle of *Rana sylvatica* in response to 24 Hr anoxia and 4 Hr recovery as determined by western immunoblotting. Data are mean ± SEM (*N* = 3–5 independent protein isolations from different animals). Other information as in Fig. 2.

Relative protein expression levels of anti-apoptotic proteins Bcl-2, p-Bcl-2 (S70), and Bcl-xL were measured using western immunoblotting in skeletal muscle tissues (Fig. 5). p-Bcl-2 (S70) and Bcl-xL showed significant downregulation in expression compared to the normoxic control conditions (*p* < 0.05). On the contrary, Bcl-2 showed increased expression during 4 Hr recovery compared to control.

x-IAP and c-IAP, that are responsible for inhibiting the function of pro-apoptotic proteins Caspase 3, 6, 7, showed no difference in expression compared to the control conditions (*p* < 0.05; Fig. 6).

**Figure 7:**
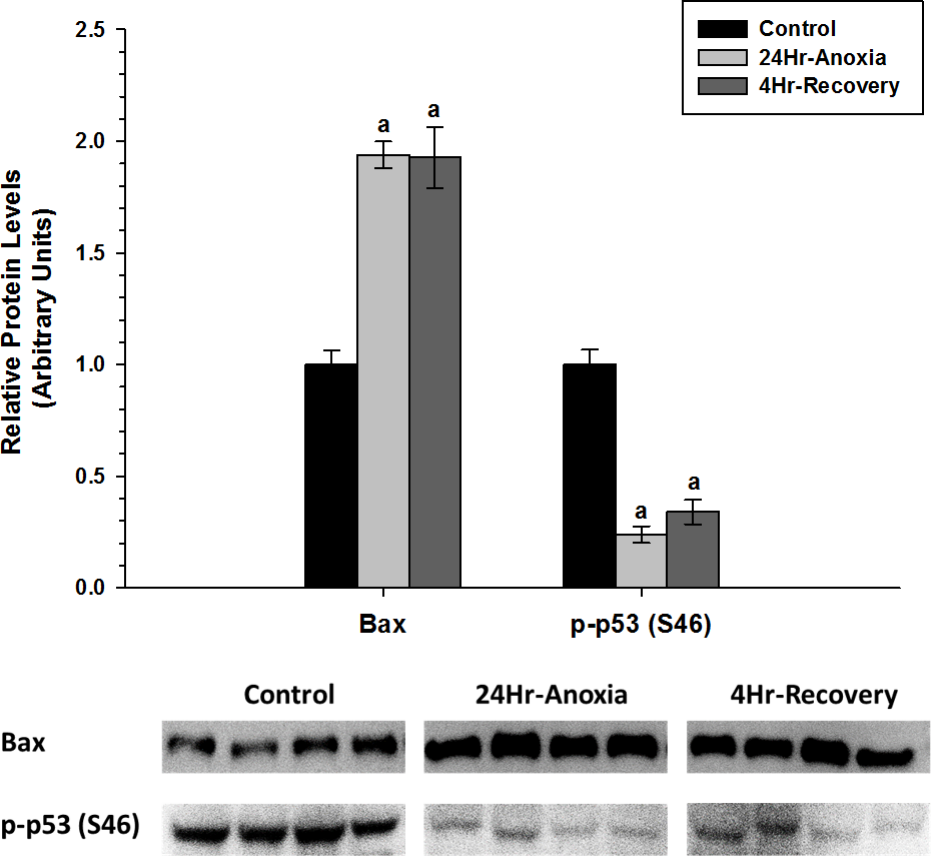
Relative changes in the protein expression levels of the pro-apoptotic proteins Bax and p-P53 (S46) in the skeletal muscle of *Rana sylvatica* in response to 24 Hr anoxia and 4 Hr recovery as determined by western immunoblotting. Data are mean ± SEM (*N* = 3–5)independent protein isolations from different animals). Other information as in Fig. 2.

In addition, relative protein expression levels of pro-apoptotic proteins Bax and p-p53 (S46) were measured using western immunoblotting in skeletal muscle (Fig. 7). Bax showed a two-fold upregulation whereas p-p53 (S46) showed a significant downregulation in expression upon exposure to 24 Hr anoxia and 4 Hr recovery conditions (*p* < 0.05).

## Discussion

The North American Wood frog, *R. sylvatica*, can survive sub-zero temperatures by utilizing a strategy called freeze-tolerance, in which 65–70% of the total body water is converted into extracellular ice (Storey & Storey, 1984). One of the main challenges of freeze-tolerance is the onset of anoxia and ischemia, such that freezing of the blood plasma cuts off oxygen and nutrient delivery to other organs and removal of waste is interrupted. In addition, having 65–70% of the body frozen means that metabolic rate will be substantially low as ATP is only produced through anaerobic means (i.e., glycolysis) and the limited amount of ATP produced is used primarily to support basal metabolic functions and maintain only the necessary molecular pathways for survival such as antioxidant defenses (Joanisse & Storey, 1996; Dawson, Katzenback & Storey, 2015) and the heat shock protein (HSP) response. Ischemia-reperfusion events generally lead to oxidative damage as re-oxygenation results in a rapid increase in reactive oxygen species in the intracellular space that can damage cellular macromolecules. Therefore, both energy conservation and cytoprotective strategies need to be equally coordinated to ensure long-term survival in the freeze tolerant frog that has been shown to survive several weeks of continuous freezing (Layne & First, 1991) and can survive two days of exposure to nitrogen gas at 5 °C (Holden & Storey, 1997).

As such, the present study explores the apoptotic/pro-survival pathways to assess whether anti-apoptosis is part of the hypometabolic response that is aimed at conserving energy and a possible cytoprotective mechanism used to enhance long-term survival and recovery. In particular, the relative expression levels of pro-survival targets involved in the inhibition of Mitochondrial Outer Membrane Permeabilization (MOMP) (Bcl-2, p-Bcl-2 (S70), and Bcl-xL) and inhibition of caspases 3, and 7 (c-IAP and x-IAP), and two main pro-apoptotic proteins (Bax and p-p53 (S46)) were measured by western immunoblotting, comparing normoxic-control conditions to 24 Hr anoxic, and 4 Hr oxygen recovery in liver and skeletal muscle.

Taken together, the data reveals that there is tissue-specific regulation of the anti-apoptotic proteins during both 24 Hr anoxic and 4 Hr recovery conditions in *R. sylvatica*. In liver, the anti-apoptotic targets that inhibit MOMP, the Bcl family of proteins, show differential expression patterns (Fig. 2). Bcl-xL in particular increased in expression by 0.5–2 fold in response to 24 Hr anoxia and 4 Hr recovery, respectively, while Bcl-2 and p-Bcl-2 (S70) significantly decreased (*p* < 0.05). Competitive dimerization between pro and anti-apoptotic protein targets determines cell survival or death. Bcl-2 and Bcl-xL can dimerize with the pro-apoptotic protein Bax (Chittenden et al., 1995; Sedlak et al., 1995; Reed, 1997; Ding et al., 2014), and according to Yang et al. (1995), 50% reduction in the formation of Bcl:Bax heterodimers and increase in Bax:Bax homodimerization facilitate cell death. An increase in Bcl-xL expression during 24 Hr anoxia and 4 Hr recovery in the wood frog would enhance heterodimerization between Bcl-xL and Bax and thereby inhibit apoptosis.

Although the physiological relevance of Bcl-2 phosphorylation is still under investigation, there is strong evidence suggesting that phosphorylation of Bcl-2 at S70 leads to loss of anti-apoptotic function (Ito et al., 1997; Deng et al., 2003). Energy conservation is of utmost importance during times of long-term anoxia and ischemia, thus liver could be monopolizing Bcl-xL to promote pro-survival of the hepatocytes as opposed to using both Bcl-2 and Bcl-xL. Therefore, the decrease in p-Bcl-2 levels seen in the liver along with increased expression of Bcl-xL protein could be a sign of enhanced cell survival.

Rouble et al. (2013) suggested that increases in IAP protein levels shown in hibernating ground squirrels may promote cell survival against ER-induced apoptosis and function as an antagonistof cell death. Furthermore, in several apoptotic models, severe hypoxia and anoxia have been shown to upregulate IAP expression and facilitate cell survival independent of other cytoprotectant mechanisms (Dong et al., 2001; Dong, Wang & Venkatachalam, 2003; Hamanaka et al., 2009). Similarly, there was an upregulation of c-IAP in response to 24 Hr anoxia in the wood frog (Fig. 3). This could suggest a possible inhibition of caspase 3 and 7 activity and further repression of the apoptotic pathway in the liver.

In opposition to anti-apoptotic proteins, pro-apoptotic proteins steer cells toward death once the cells have been exposed to injury and are deemed irreparable. However, during metabolic rate depression in which ATP is in limited supply, cellular components and macromolecules will not be robustly regenerated, and the cell cycle is strongly suppressed (Zhang & Storey, 2012). The anti-apoptotic pathway could play an essential role in cytoprotection similar to the increase in antioxidant defenses (Dawson, Katzenback & Storey, 2015) aimed at preserving the existing cellular components during long term anoxia and oxygen recovery phase. In the liver, pro-apoptotic proteins Bax and p-p53 (S46) did not show an increase in expression during both 24 Hr anoxia and 4 Hr oxygen recovery conditions (Fig. 4). Furthermore, Akt has been shown to inhibit programmed cell death through increased phosphorylation of p21 and FOXO1, two transcription factors when phosphorylated at T145 and S256 respectively promote cell survival, during 24 Hr freezing and 8 Hr thaw in the liver of wood frogs (Zhang & Storey, 2012; Zhang & Storey, 2013). This could further suggest that apoptosis may not be fully activated in the liver.

Liver has been shown to contain approximately 1,000 mol/gww of glycogen (Storey & Storey, 1996) prior to the onset of freezing and play an important role in regulating both cryoprotection and energy maintenance. One of the main biochemical adaptations that support freeze tolerance and survival in wood frogs is the release of glucose as a cryoprotectant that regulates cell volume during extracellular ice formation (Storey & Storey, 1986). Therefore, very high concentrations of glucose are synthesized and rapidly distributed by the liver within minutes of the first onset of freezing; blood and liver glucose rise by 3.3–6.6 fold respectively, while overall glucose levels in core organs can rise to 150–300 mol/gram wet weight (gww) (Storey & Storey, 1986; Storey, Bischof & Rubinsky, 1992; Storey & Mommsen, 1994). Thus, enhanced mechanisms of cell survival could be put in place by the wood frog to preserve this essential organ during times of low oxygen availability.

In the skeletal muscle, p-Bcl-2 (S70) and Bcl-xL showed a significant decrease during anoxic and recovery conditions, while Bcl-2 showed a significant increase in expression during 4 Hr recovery compared to the control and 24 Hr anoxia (Fig. 5). Hockenbery et al. (1993) found that Bcl-2 prevents cells from H_2_O_2_ and oxidative stress-induced deaths. A substantial portion of the oxidative damage tends to occur not during the anoxic period but rather during the reperfusion phase when oxygen is reintroduced into the system. The sudden increase in oxygen radicals during reperfusion can temporality overwhelm the cellular antioxidant system and impose irreversible damage to DNA, proteins, lipids, and alter the viability and integrity of cells (Storey & Storey, 2004a). Bcl-2 was shown to be involved with fine-tuning the balance between mitochondrial oxygen consumption for energy production and ROS generation by modifying mitochondrial respiration to fulfill energy demands without inducing harmful increases in ROS (Chen & Pervaiz, 2007). In addition, Bcl-2 may be important in maintaining membrane lipid integrity by suppressing ROS damage (Kane, Sarafin & Auton, 1993) and has been implicated in protein and ion transport across cell membranes (Liu et al., 1996; Kluck et al., 1997; Lam et al., 1994). Ion channel regulation in particular plays an integral part in controlling a cell’s susceptibility to death and as such Bcl-2 and Bcl-xL have been shown to form ion channels on synthetic lipid membranes (Minn et al., 1997).

Additionally, increased expression levels of Bcl-2 and significant decrease in p-Bcl-2 (S70) could indicate an enhanced heterodimerization between Bcl-2 and Bax, thus delaying Bax translocation to the mitochondria (Chittenden et al., 1995; Sedlak et al., 1995; Reed, 1997) to facilitate apoptosis. According to Basu & Haldar (1998), phosphorylation at S70 can disrupt the Bcl-2:Bax heterodimerization to a certain extent and facilitate homodimerization of Bax to each other. Therefore, a decrease in p-Bcl-2 (S70) levels in 24 Hr anoxic and 4 Hr recovery could be a preventative mechanism that ensures Bcl-2 exists in a hypophosphorylated state to maintain cell survival.

The upregulation of pro-apoptotic protein Bax in the skeletal muscle suggests that apoptosis could be further inhibited by another mechanism. Previous studies have found that HSPs can inhibit apoptosis. For example, Hsp27, Hsp70 and Hsp90 have been shown to bind to Apaf-1and reduce caspase activation (Beere et al., 2000; Pandey et al., 2000). Hsp27 can also inhibit the release of cytochrome c from the mitochondria (Gorman et al., 2005), and bind directly to cytosolic cytochrome c, sequestering it from Apaf-1 (Garrido et al., 1999). Hsp60 has also been shown to heterodimerize with Bax and prevent Bax translocation into the mitochondria from the cytosol (Gupta & Knowlton, 2002). Additionally, the significant decrease in p-p53 (S46) levels during 24 Hr anoxic and 4 Hr thaw by 0.5-fold could further suggest that apoptosis is possibly restricted in the skeletal muscle as p53 phosphorylation at S46 residue is known to induce a pro-apoptotic response (Fig. 7) (Vousden & Lu, 2002). Furthermore, Wang et al. (2006) investigated the relationship between cytoprotection and apoptosis and reported that mitochondrial-driven apoptotic events such as Bax accumulation, cytochrome c release, and p53 activation were substantially suppressed during hypoxia and anoxia to facilitate cell viability and survival.

Overall, the current data suggests a tissue-specific regulation of the anti-apoptotic pathway, which could be attributed to distinctive oxidative challenges to liver and skeletal muscle in the wood frog during long-term anoxia and recovery. Furthermore, the interplay between anti-apoptotic proteins Bcl-2, p-Bcl-2(S70), Bcl-xL, c-IAP, and x-IAP and pro-apoptotic proteins Bax, and p53 may determine cell fate (Basu & Haldar, 1998), and as suggested by Rouble et al. (2013) activation of the anti-apoptotic pathway may be central to energy conservation during hypometabolism and play a cytoprotectant role that ensures long-term cell and macromolecular viability during 24 Hr anoxic and 4 Hr aerobic recovery conditions.

## Additional Information and Declarations

### Data Availability

The following information was supplied regarding data availability: Raw ECL and Coomassie data corresponding to the study can be provided upon request. Please email the corresponding author.
